# Age Related Differences in Dynamics of Specific Memory B Cell Populations after Clinical Pertussis Infection

**DOI:** 10.1371/journal.pone.0085227

**Published:** 2014-01-13

**Authors:** Inonge van Twillert, Jacqueline A. M. van Gaans-van den Brink, Martien C. M. Poelen, Kina Helm, Betsy Kuipers, Maarten Schipper, Claire J. P. Boog, Theo J. M. Verheij, Florens G. A. Versteegh, Cécile A. C. M. van Els

**Affiliations:** 1 Centre for Immunology of Infectious Diseases and Vaccines, National Institute for Public Health and the Environment (RIVM), Bilthoven, The Netherlands; 2 Department of Statistics, Mathematical Modelling and Data Logistics, National Institute for Public Health and the Environment (RIVM), Bilthoven, The Netherlands; 3 InTraVacc, Bilthoven, The Netherlands; 4 Julius Center Health Sciences and Primary Care, University Medical Center Utrecht, Utrecht, The Netherlands; 5 Department of Pediatrics, Groene Hart Ziekenhuis, Gouda, The Netherlands; Universidad Nacional de La Plata, Argentina

## Abstract

For a better understanding of the maintenance of immune mechanisms to *Bordetella pertussis* (Bp) in relation to age, we investigated the dynamic range of specific B cell responses in various age-groups at different time points after a laboratory confirmed pertussis infection. Blood samples were obtained in a Dutch cross sectional observational study from symptomatic pertussis cases. Lymphocyte subpopulations were phenotyped by flowcytometry before and after culture. Memory B (B_mem_) cells were differentiated into IgG antibody secreting cells (ASC) by polyclonal stimulation and detected by an ELISPOT assay specific for pertussis antigens pertussis toxin (Ptx), filamentous haemagglutinin (FHA) and pertactin (Prn). Bp antigen specific IgG concentrations in plasma were determined using multiplex technology. The majority of subjects having experienced a clinical pertussis episode demonstrated high levels of both Bp specific IgG and B_mem_ cell levels within the first 6 weeks after diagnosis. Significantly lower levels were observed thereafter. Waning of cellular and humoral immunity to maintenance levels occurred within 9 months after antigen encounter. Age was found to determine the maximum but not base-line frequencies of B_mem_ cell populations; higher levels of B_mem_ cells specific for Ptx and FHA were reached in adults and (pre-) elderly compared to under-fours and schoolchildren in the first 6 weeks after Bp exposure, whereas not in later phases. This age effect was less obvious for specific IgG levels. Nonetheless, subjects' levels of specific B_mem_ cells and specific IgG were weakly correlated. This is the first study to show that both age and closeness to last Bp encounter impacts the size of Bp specific B_mem_ cell and plasma IgG levels.

## Introduction

*Bordetella pertussis* (Bp) causes the respiratory infectious disease ‘whooping cough’ (pertussis) which is especially hazardous for neonates. Wide spread introduction of vaccination programmes in the 1950's resulted in a considerable decline in the incidence and severity of pertussis through protection of vaccinees and subsequent herd-immunity [1]–[3]. Nevertheless pertussis has remained endemic and in the last decade a mounting number of pertussis notifications and hospitalized cases among adolescents, adults and elderly has been observed in well-vaccinated populations [4]–[6]. These higher incidence rates are likely caused by a combination of factors. Firstly, primary protective immunity after either vaccination or natural infection is gradually lost within 5 to 10 years [7]–[11]. Secondly, multiple studies examining the genomic content of circulating *B. pertussis* isolates have described pathogen adaptation to the vaccinated host [12]–[20]. Lastly, the reduction of Bp circulation as a result of extensive vaccination coverage has led to less natural boostering of acquired immune mechanisms in vaccinees, leading to an increasing group of pertussis-susceptible adults. These have now become a source of transmission to vulnerable groups such as elderly and infants too young to be (fully) vaccinated. Both humoral and cellular immune mechanisms are associated with protection from pertussis disease [21]. Pertussis infections as well as vaccination initially induce high levels of antibodies against pertussis specific antigens. The detection of specific serum antibodies is the most widely applied method to investigate host immunity as well as the seroprevalence of pertussis [22]–[25]. Despite evidence for the contribution of antibody levels to all major vaccine antigens in resistance against pertussis [26]–[29], no serologic correlates of protection exist. In addition to antibody levels, memory B (B_mem_) cells and CD4^+^ T cells have been identified to be important for protection against pertussis [30]–[32]. In the absence of detectable serum antibodies, protection is often still maintained [33], [34] implying a role for other key players of the immune system such as circulating B_mem_ cells that can rapidly proliferate and differentiate into antibody producing cells (ASC) upon encounter with antigen [35]–[37]. Thus far, understanding on the prevalence of human pertussis specific B_mem_ cells has been mostly limited to vaccinated children. Hendrikx *et al* found pertussis specific B_mem_ cells in three to nine year olds despite waning IgG-Ptx antibody levels [38]. Pertussis booster vaccination was associated with a temporary rise of circulating B_mem_ cells [39]–[41]. However, little is known about B_mem_ cell responses across age groups. The capability of the B cell compartment to respond to pertussis antigens may depend on age-related constrictions of the immune system, ranging from immatureness in new-borns to immunosenescence in elderly [42], [43], but also on the circumstances of antigen encounter. The aim of the present study was to gain insight into the dynamic range of pertussis specific IgG and B_mem_ cell responses induced by symptomatic pertussis infection in various age-groups. Both the effect of age and time elapsed since the pertussis infection on the quantitative outcome of the B cell response were studied. Patterns observed in the B_mem_ cell compartment were analysed in relation to humoral responses.

## Subjects and Methods

### Ethics Statement

This study was conducted according to the principles expressed in the Declaration of Helsinki. The study was approved by the accredited Review Board STEG (Stichting Therapeutische Evaluatie Geneesmiddelen) and is currently managed by the METC UMC Utrecht (Medisch Ethische Toetsingscommissie Universitair Medisch Centrum Utrecht) (CCMO nr: NL16334.040.07). Practicability of the study in the collaborating hospitals was accorded by their Review Boards. All participants provided written informed consent for the collection of samples, the usage of a completed questionnaire regarding clinical symptoms and vaccination history, and the subsequent immunological analysis. Informed written consent for minor participants was provided by both parents of participants.

### Study population

For this study blood samples were obtained from one hundred seventy four participants as part of a cross-sectional observational study investigating pertussis specific immunity in the general Dutch population. The participants investigated herein were male (n =  67) and female (n = 107) pertussis patients, aged between 10 months – 83 years (median 15 years), recruited between January 2008 and December 2012 at a known time interval after their laboratory confirmed symptomatic pertussis infection. Introduction of an acellular pertussis (aP) pre-school booster in the Netherlands was in 2001, the replacement of the whole cell pertussis vaccine (wP) series at 2, 3, 4 and 11 months of age with aP vaccination took place in 2005. For analysis, subjects were classified into five age groups, depending on both age and history of childhood pertussis vaccination: under-fours (u4s), schoolchildren (sch), adolescents (ado), adults (adu), and (pre-) elderly (eld) (for details see Table 1). Subjects were also stratified based on the time period elapsed between last known date of exposure to pertussis antigen and date of blood sampling (*time after exposure)*: [≤1.5 months], [1.6–8.9 months] and [≥ 9.0 months]. In 142 cases last known date of exposure was their date of diagnosis. For 32 out of 174 cases (in the under-fours, children and adolescent groups), last known date of exposure to pertussis antigen was their date of last vaccination, since they had their pertussis episode before the age of 4 and had received one or more pertussis vaccinations thereafter. In the same groups, 5 participants had received no pertussis vaccination at all for religious or ulterior motives. All (pre) elderly were born before pertussis vaccination was implemented in the national immunization programme. Serological exclusion criteria were used for eligibility in the [≥ 9.0 months] *time after exposure* groups. Excluded from these groups were cases with Ptx specific IgG levels >62.5 IU/ml and *time after exposure* >24 months, indicative for an additional, more recent non-diagnosed exposure [25]. Nine subjects recruited ≤1.5 months after their diagnosis donated a second blood sample 3.0–4.7 (median 3.8) years after diagnosis (for details see table 2), generating a total number of 183 blood samples. For cross-sectional evaluation of *time after exposure* effects, acute samples from these nine subjects were excluded from biostatistical analyses.

**Table 1:** Cohort description.

CohortCovariate	Under-fours			School children			Adolescents			Adults			(Pre) elderly		
Abbreviated to	u4s			sch			ado			adu			eld		
*Time since exposure* cohort	≤ 1.5 months	1.6 – 8.9 months	≥ 9.0 months	≤ 1.5 months	1.6 – 8.9 months	≥ 9.0 months	≤ 1.5 months	1.6 – 8.9 months	≥ 9.0 months	≤ 1.5 months	1.6 – 8.9 months	≥ 9.0 months	≤ 1.5 months	1.6 – 8.9 months	≥ 9.0 months
Number of participants (n)	9	5	6	12	12	21	12	6	18	27	9	12	20	3	11
Sex; male/female (n)	5/4	4/1	2/4	11/1	4/8	15/6	6/6	3/3	10/8	19/8	7/2	7/5	11/9	2/1	5/5
Age; median/range (yrs)	2.3/1.0–3.7	1.1/0.8–1.6	3.3/2.0–3.9	7.0/4.2–10.0	7.4/4.2–10.3	8.1/5.6–13.1	12.9/11.1–18.2	14.4/11.0–19.2	14.0/11.0–18.7	47.9/22.4–54.5	42.9/28.3–56.6	38.4/21.9–59.5	66.5/58.2–78.9	63.5/58.5–72.9	67.1/61.7–83.4
History of P vaccination^1^	aP(1–4)			a or wP(1–4) + aP(5)			wP(1–4)			wP(1–4)			no		
Compliant with NIP schedule (n)	9	4	6	12^3^	12^4^	18^5^	12	6	17	27	9	12	N.A.		
Voluntarily non-vaccinated (n)	-	1	-	-	-	3	-	-	1	-	-	-	N.A.		
Time after exposure^2^; geomean/range (months)	0.8/0.2–1.5	3.0/1.6–6.8	23.4/11.0–34.5	0.9/0.4–1.5	2.3/1.6–6.5	55.9/20.8–108.6	0.8/0.5–1.2	1.9/0.9–8.0	71.6/16.4–192	0.8/0.3–1.5	3.0/1.6–8.0	45.5/14.5–182	0.7/0.4–1.2	3.3/1.6–5.6	31.0/12.5–61.9

^1^ aP and wP; acellular or whole cell pertussis vaccine; ^2^ ‘time since exposure’ is either time between blood sampling and episode of symptomatic pertussis infection or time between blood sampling and last (aP or wP) vaccination, following an earlier episode of symptomatic pertussis; ^3^ all had wP(1–4) as primary series except two participants who had aP(1–4) and 1 participant who had wP(1–3), aP(4);^ 4^ all had wP (1–4) as primary series except three participants who had aP(1–4) and 1 participant who had wP(1–3), aP(4); ^5^ all had wP (1–4) as primary series except two participants who had aP(1–4) and 1 participant who had wP(1–3), aP(4).

**Table 2:** Description of subjects sampled for longitudinal data.

Subject	Sex (male/female)	Age at diagnosis (years)	Age-group
HU16401	male	18.2	adolescents
GH16201	female	26.9	adults
HU15501	female	49.6	adults
SP16301	male	52.8	adults
HU20701	male	58.2	(pre) elderly
HU21101	female	61.9	(pre) elderly
HU20601	female	63.5	(pre) elderly
HU20101	male	64.3	(pre) elderly
HU21001	female	67.0	(pre) elderly

### Pertussis antigens

P.69 pertactin (Prn) was recombinantly expressed and purified from *E. coli* as described previously [44]. Pertussis Toxin (Ptx) and Filamentous Hemagglutinin (FHA) were both obtained from Kaketsuken, Japan.

### Isolation of peripheral blood mononuclear cells and plasma

Venous blood samples were taken by a trained nurse during home-visits and collected in vacutainer cell preparation tubes (CPT) containing sodium citrate (Becton Dickinson Biosciences, USA (BD)). Peripheral blood mononuclear cells (PBMC) and plasma were isolated from sample tubes by density gradient centrifugation according to manufacturer's instruction and wash steps using RPMI-1640 (Gibco, USA) with 10% fetal bovine serum (FBS; Hyclone, USA). Isolated PBMC were frozen in 90% FBS (Hyclone) and 10% dimethyl sulfoxide (DMSO, Sigma) at −80°C overnight and stored at −135°C until testing. Plasma was stored at −80°.

### Facs analysis of B cells

PBMC before and after 5 days of B cell differentiation were analysed by multicolour flow cytometry. Cells were incubated for 20–30 minutes with a mixture of PE labelled anti-human CD138, PerCP-Cy5.5 labelled anti-human CD19 and APC labelled anti-human CD27 (eBiosciences) and were subsequently analysed using the FACS Canto and FlowJo software. Results are depicted in bar charts as percentages of lymphocytes or of CD19^+^ B cells as indicated. The bars represent geometric means (GM) with 95% confidence interval (C.I).

### B cell stimulation and B-cell ELIspot assay

The B cell stimulation assay and the pertussis specific B-cell ELIspot assay for human PBMC was developed based on methods described elsewhere [45] with minor modifications.

#### B cell stimulation

PBMC were quickly thawed at 37°C, washed in RPMI 1640 containing 10% FBS, centrifuged and subsequently cultured for 5 days at 37 °C and 5% CO_2_ in AIM-V with albumax (Gibco, Invitrogen) supplemented with 10% FBS (Hyclone) and 50 µM β-mercaptoethanol (AIM-V+) at a concentration of 2×10^6^ cells/ml. To differentiate memory B cells into Antibody Secreting Cells (ASC) the medium was further supplemented with an optimized cocktail of polyclonal and specific stimuli, final concentrations of which were: 3 µg/ml CpG: ODN2006 (PTO modified 5′-TCG TCG TTT TGT CGT TTT GTC GTT- 3′) Isogen Life Sciences, Maarssen, The Netherlands), 10 ng/ml IL-10 (Calbiochem), 10 ng/ml IL-2 (Miltenyi Biotec, Auburn, USA) and 10 ng/ml of the pertussis antigens Ptx, FHA and Prn.

#### B-cell ELIspot assay

96-well high-protein-binding multiscreen HTS plates (Millipore, UK) were coated overnight at 4 °C (50 µl/well) with 50 µl PBS containing either 30µl/ml PTX, 20 µg/ml FHA or 30 µg/ml Prn for enumerating antigen specific ASC and 10 µg/ml goat-anti human-IgG (MP Biomedicals, Ohio, USA) to measure total IgG secreting cells as a positive control. Plates were blocked (200 µl/well AIM-V+ for 2 hours, 37 °C) and 2-fold serially diluted cultured PBMC suspensions were incubated at a starting concentration of 2*10^5^ cells/well in AIM-V+ in duplicate wells overnight (100 µl/well, 37 °C, 5% CO_2_). Subsequently, plates were washed with 0.05% Tween-20 in PBS (PBS-TW) and incubated with 1: 5000 alkaline phosphatase-conjugated goat-anti-human IgG (Sigma, UK) in 0.01% Tween20, 1% goat serum (ICN Biomedicals, USA) in PBS for 2–4 hours at 37 °C. After incubation, plates were washed with PBS-TW followed by washing with PBS before development with a ready-to-use 5-bromo-4-chloro-3-indolylphosphate/nitrobluetetrazolium (BCIP/NBT, KPL, USA) solution for 5–30 minutes at RT. Then plates were washed with water and allowed to dry. Spots, representing ASC, were counted using an Immunoscan^tm^ (CTL, Germany). Spots from at least two countable cell dilutions were used and expressed as geometric mean ASC per 10^5^ plated cells. Negative control wells (wells not coated but incubated with cells in AIM-V+) were used to calculate background. These background spot numbers were subtracted from the antigen specific ASC. Results are depicted using box-and-whisker plots, with the whiskers representing the 5–95% confidence interval.

### Serology

To measure levels of specific human IgG antibodies against pertussis antigens Ptx, FHA, and Prn, we employed a human multiplex immunoassay (MIA) based on luminex technology developed by van Gageldonk *et al*.[46]. Briefly, for each antigen, fluorescently labeled microspheres from a distinct bead region (Bio-Rad Laboratories, Hercules, USA) were activated at 12.5*10^6^ beads/ml with 5 mg/ml EDC and 5 mg/ml NHS (Pierce,USA) in PBS (Invitrogen, USA) and subsequently washed and resuspended in PBS containing 5 µg antigen/6.25*10^6^ activated beads/250 µl. Antigens were allowed to bind (2 hours, RT, 25 rpm) and beads were subsequently washed and stored in PBS-0.05% sodium azide-1% BSA (Sigma-Aldrich, USA) at 4 °C in the dark until use. We made use of the international standardized pertussis reference serum WHO NIBSC 06/140. Eight steps of 3-fold dilutions of the reference serum (1/30 to 1/65610) in PBS-3% BSA-0.1% Tween-20 (Merck, Germany) (PBS-T20-BSA). Sera samples were prepared in two dilutions; 1/300 and 1/3600 in PBS-T20-BSA. Each dilution of serum (25 µl) was mixed with an equal volume of conjugated microspheres (4000 beads/region/well) in a 96-well multiscreen HTS filter plate (Low-protein binding, Millipore, USA) and incubated 45 minutes at RT. Then, beads were washed (PBS), and bound antibodies were detected by Goat anti-human IgG, R-PE (Jackson ImmunoResearch, USA) in PBS. Analysis was performed with a Bio-Plex 100 in combination with Bio-Plex Manager software version 5 (Bio-Rad Laboratories, USA). For each analyte, fluorescent intensity (FI) was converted to IU/ml by interpolation from a 5-parameter logistic standard curve (log-log) for every bead region/standard. Data were expressed in IU/ml in box-and-whisker plots on a log10 scale, with whiskers representing 5-95% C.I.

### Statistical analysis

**Figure 1:**
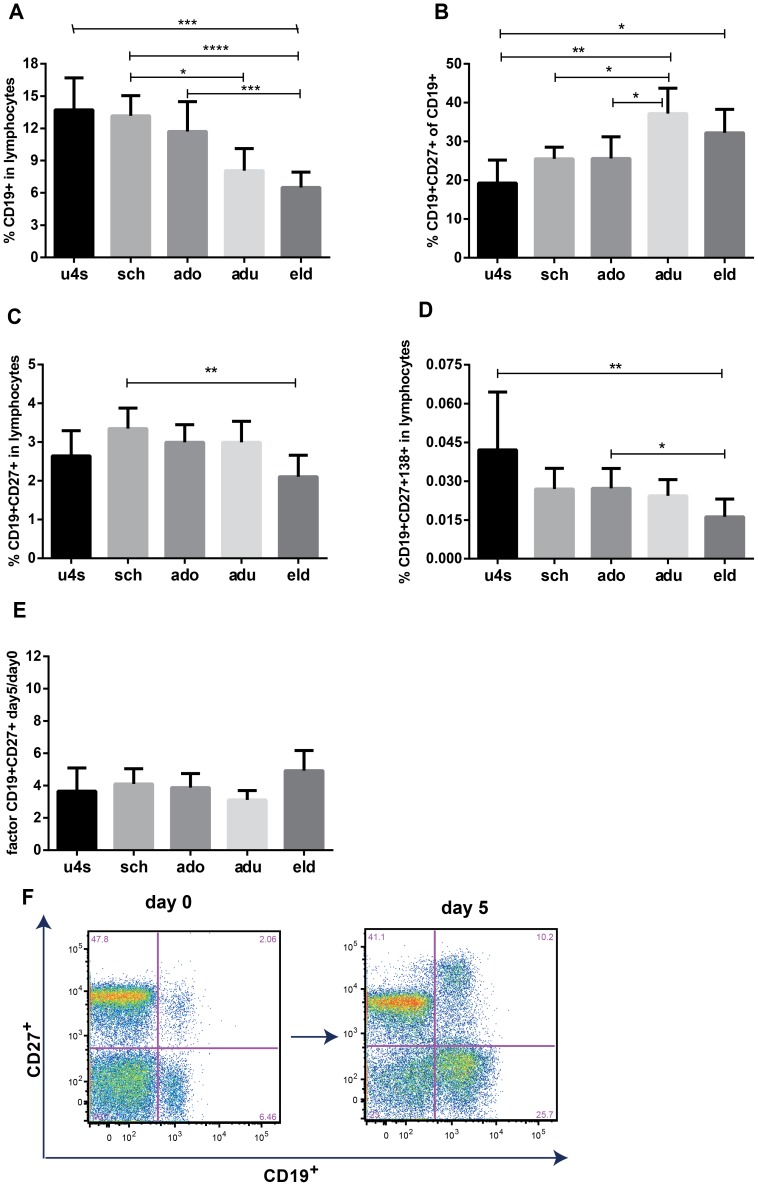
Flow cytometry analysis of B cell populations before and after culture. Shown are bar charts of B cell frequencies of our five age groups with geometric means and 95% CI. Graphs show relative frequencies of total B cells (A) as well as memory B cells (C) and plasma cells (D) as percentage of all lymphocytes and memory B cells as percentage of all CD19^+^ B cells (B) per age group. The factor of enrichment of memory B cells due to in vitro stimulation is likewise shown per age-group (E) and exemplified by one donor in two flow cytometry dot plots (F). Age groups are designated as follows; u4s (under-fours), sch (schoolchildren), ado (adolescents), adu (adults) and eld ((pre-) elderly).

**Figure 2:**
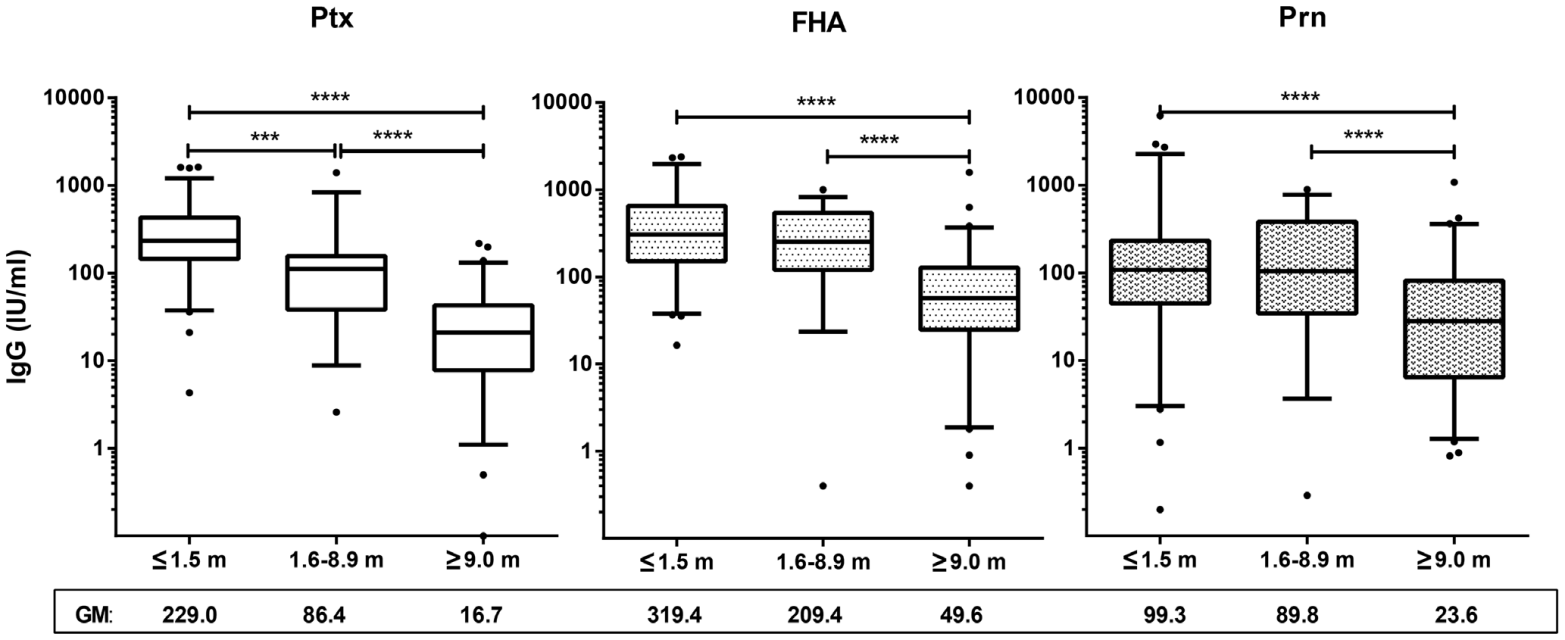
Pertussis specific IgG levels decrease with time. Box plots of specific IgG concentrations of Ptx (left), FHA (middle) and Prn (right) in symptomatic cases in *acute* (≤1.5 months), *intermediate* (1.6–8.9 months) and *maintenance phase* (≥9 months) after the last known immunizing event. Box plots show first and third quartiles and medians and whiskers represent 5–95% CI. Below the x-axis geometric mean values are given for each group. ∧ =  p<0.1; * = p<0.05; ** =  p< 0.01; *** =  p<0.001, **** =  p<0.0001

**Figure 3:**
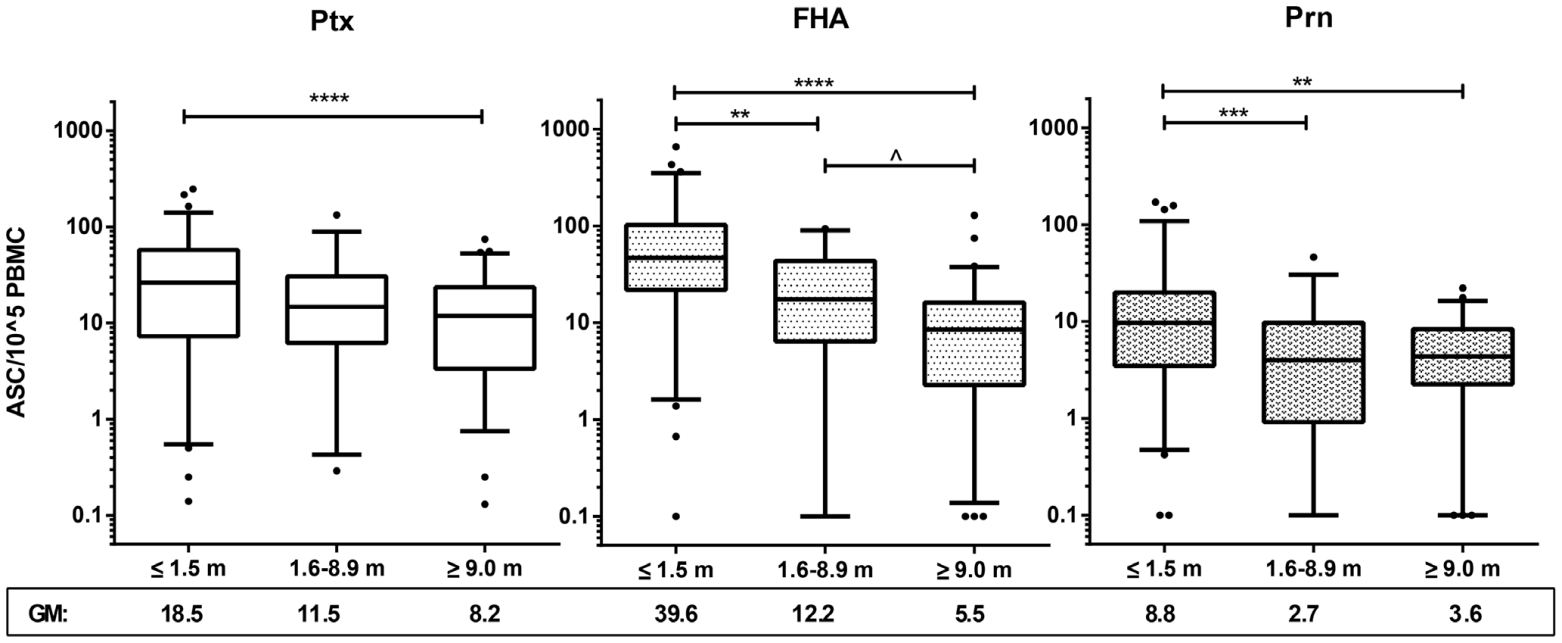
Pertussis specific levels of B_mem_ decrease with time. Box plots of specific B_mem_ frequencies of Ptx (left), FHA (middle) and Prn (right) in symptomatic cases in *acute* (≤1.5 months), *intermediate* (1.6–8.9 months) and *maintenance phase* (≥9 months) after the last known immunizing event. Box plots show first and third quartiles and medians and whiskers represent 5–95% CI. Below the x-axis geometric mean values are given for each group. ∧ =  p<0.1; * = p<0.05; ** =  p< 0.01; *** =  p<0.001, **** =  p<0.0001

**Figure 4:**
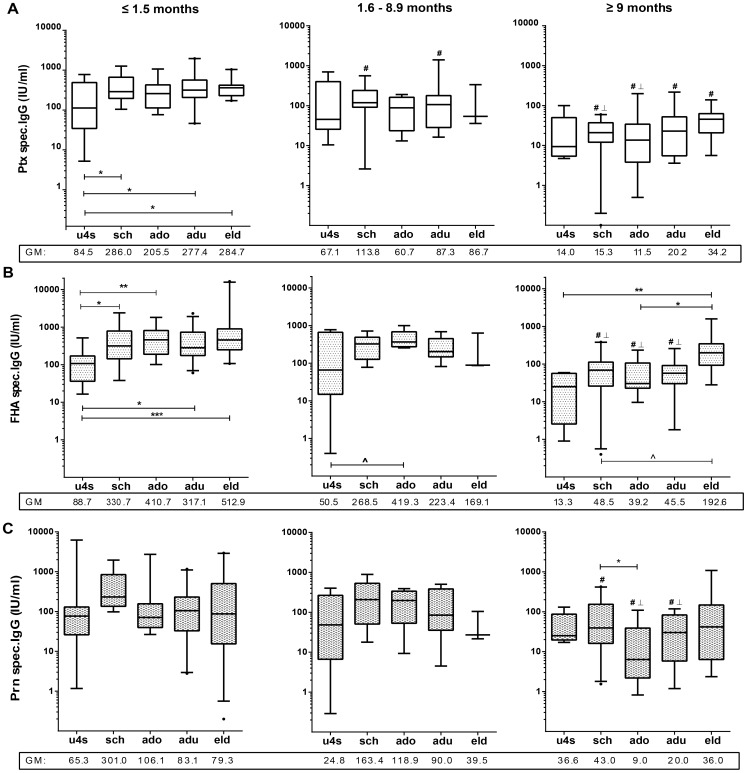
Comparing pertussis specific IgG levels between age-groups and per *time after exposure*. Box plots representing age stratified levels of Ptx-, (A) FHA- (B) and Prn- (C) specific IgG concentrations in *acute* (≤1.5 months), *intermediate* (1.6–8.9 months) and *maintenance phase* (≥9 months) after last known immunizing event. Age groups are designated as follows; u4s (under-fours), sch (schoolchildren), ado (adolescents), adu (adults) and eld ((pre-) elderly). Below the x-axis geometric mean values are given for each group. ∧ =  p<0.1; * = p<0.05; ** =  p< 0.01; *** =  p<0.001, #  = significantly lower than same age group in *acute phase*, ⊥  = significantly lower than same age group in *intermediate phase*

**Figure 5:**
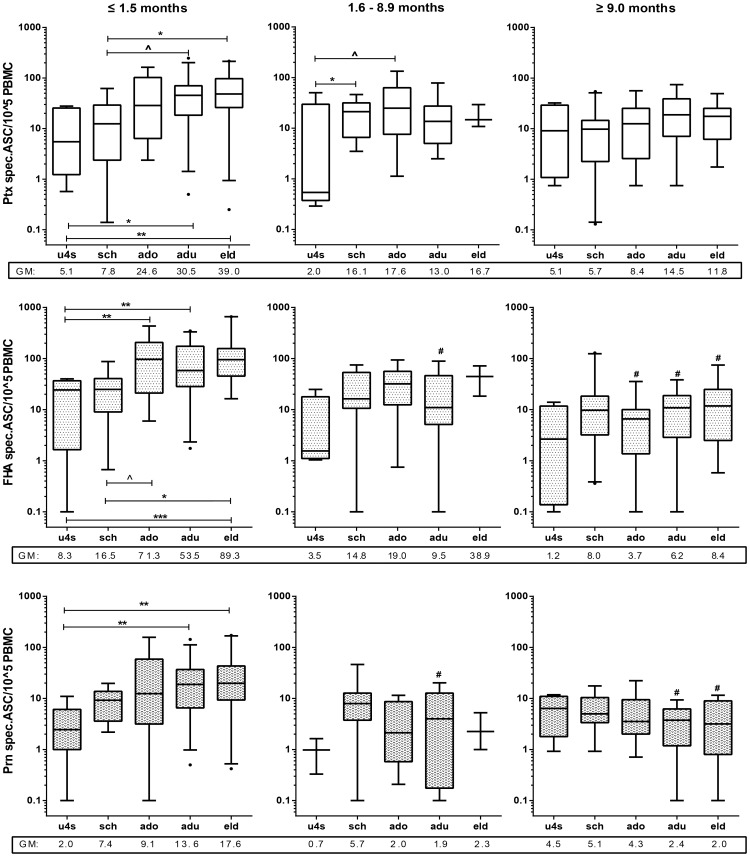
Age-related differences in pertussis specific B_mem_ cell levels in *acute phase*. Box plots representing age stratified levels of Ptx-, (A) FHA- (B) and Prn- (C) specific B_mem_ frequencies in *acute* (≤1.5 months), *intermediate* (1.6-8.9 months) and *maintenance phase* (≥9 months) after last known immunizing event. Age groups are designated as follows; u4s (under-fours), sch (schoolchildren), ado (adolescents), adu (adults) and eld ((pre-) elderly). Below the x-axis geometric mean values are given for each group. ∧ =  p<0.1; * = p<0.05; ** =  p< 0.01; *** =  p<0.001, #  = significantly lower than same age group in *acute phase*

**Figure 6:**
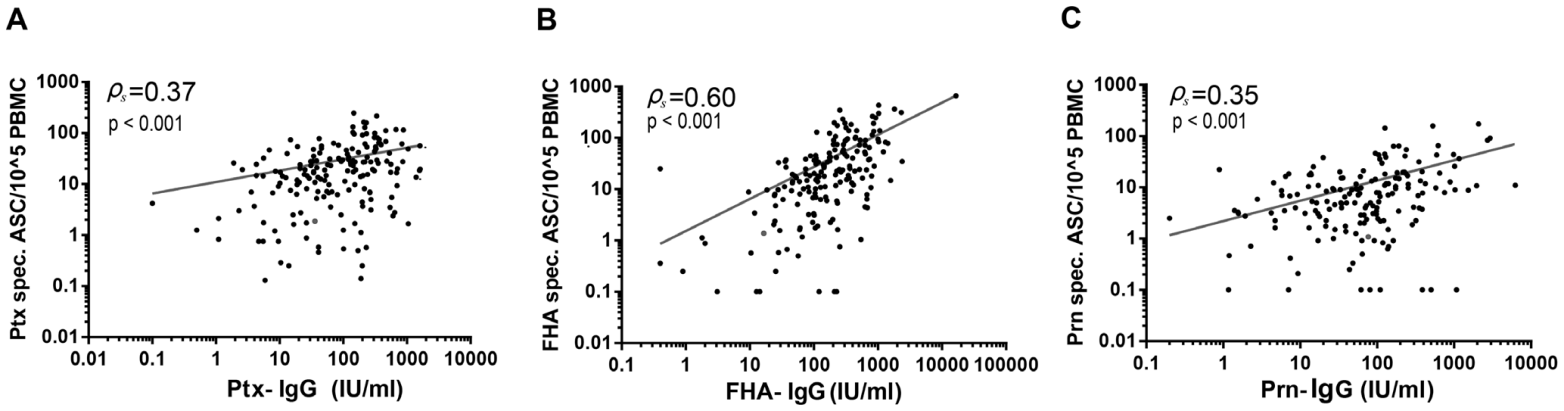
Spearman correlations between pertussis specific B_mem_ and corresponding IgG levels. Dots represent paired antigen specific B_mem_ cell frequencies and plasma IgG levels for Ptx (A), FHA (B) and Prn (C) for each participant.

For data analysis and visualization of data R software (http://www.Rproject.org/) and GraphPad Prism (GraphPad Software version 6.02) were used. Statistical significance of differences between age-groups in lymphocyte population percentages were analysed with the nonparametric Kruskal-Wallis Test and Dunn's Multiple comparison test (Fig. 1A to 1E). To determine significant differences in levels of specific B_mem_ or IgG, groups were compared using nonparametric Kruskal-Wallis Test and post-hoc Tukey's honest significance test (HSD) (Figures 2–5). P values <0.05 were considered statistically significant. Correlations between specific levels of B_mem_ and IgG concentrations were determined with Spearman's rank correlation coefficient (Figure 6). Spearman ρ values with significant p values were interpreted as follows: ρ< 0.2: none, ρ = 0.2–0.4: weak, ρ =  0.4–0.6 moderate, ρ =  0.6–0.8: strong and ρ>0.8 very strong correlation.

## Results

### Age related differences in frequencies of peripheral B cell fractions

Various studies have indicated total CD19^+^ B cells within lymphocytes to decrease and CD19^+^CD27^+^ B_mem_ cells within CD19^+^ B cells to increase with age [47]–[50]. To assess in this study any age-related trends in lymphocyte subpopulations, we phenotypically studied several B cell populations in PBMC from all participants, directly after thawing. The fraction of CD19^+^ B cells in lymphocytes was highest in the under-fours with a geometric mean (GM) percentage of 14%, and lowest in the pre-elderly with a GM of 7% (Figure 1A). In the under-fours one fifth of CD19^+^ B cells were B_mem_ cells, co-expressing CD27^+^; this fraction increased with age to one third in adults and the elderly (Figure 1B). The GM percentage CD19^+^CD27^+^ B_mem_ cells in lymphocytes was between 2.5% and 3.5% in all age-groups, except for the (pre-) elderly who had a GM of 2% (Figure 1C). This shows that although the fraction of CD19^+^CD27^+^ B_mem_ cells in the total lymphocyte population remains relatively stable in life, a higher percentage of CD19^+^ B cells has a memory phenotype in older age categories. The frequency of peripheral plasma cells, defined as CD19^+^CD27^+^CD138^+^ cells, showed a decrease with age, from (GM) 0.04% in the under-fours to 0.03% in the schoolchildren, adolescents and adults and to 0.01% of all lymphocytes in the (pre) elderly (Figure 1D). To confirm that enrichment of B_mem_ cells was achieved by our B cell stimulation assay we also analysed cells phenotypically after 5 days of culture. We found that the fraction of CD19^+^CD27^+^ B_mem_ cells was enriched 3–5 fold (Figure 1E), shown for one typical donor (Figure 1F). No significant differences in B_mem_ cell enrichment were found between age-groups.

### Magnitude of pertussis specific B cell responses after clinical infection

To investigate the overall dynamic range of the pertussis specific B cell response following clinical infection, both in the humoral and the cellular compartment, we measured Bp specific IgG concentrations as well as B_mem_ cell frequencies by MIA and B cell ELIspot assay, respectively. To identify trends related to closeness to infection, results were analysed per *time after exposure* for each of the three pertussis antigens Ptx, FHA and Prn. Clearly, both arms of the B cell response were most expanded for all antigens in the *acute phase* [<1.5 months]. Highest GM levels of specific IgG and B_mem_ cells found are 229.0, 319.4 and 99.3 IU/ml (Figure 2), and 18.5, 39.6 and 8.8 ASC/10^5^ PBMC for Ptx, FHA and Prn (Figure 3), respectively. Levels became significantly lower in the phases thereafter. The serology data showed a significantly faster decline of IgG levels specific for Ptx than those specific for FHA and Prn (Figure 2). In contrast, Ptx specific B_mem_ cell frequencies decreased more slowly than FHA and Prn specific B_mem_ cell frequencies (Figure 3). The decline of FHA and Prn-IgG responses seemed delayed when compared to their corresponding B_mem_ cell responses; B_mem_ cell frequencies reached a baseline level in the *intermediate phase* [1.5–8.9 months] (Figure 3), while IgG levels showed lowest levels in the *maintenance phase* [>9.0 months] (Figure 2). GM maintenance levels of specific IgG and B_mem_ cells were 16.7, 49.6 and 23.6 IU/ml, and 8.2, 5.5 and 3.6 ASC/10^5^ PBMC for Ptx, FHA and Prn, respectively. Antigen specific trends were not only observed in swiftness of the contraction but also in maximum size range. When comparing the B_mem_ cell responses to antigens quantitatively, FHA recruited the highest levels, Ptx had an intermediate position and Prn engaged lowest levels, both in specific IgG and in numbers of specific B_mem_ cells per 10^5^ PBMC (Figure 3). Base levels of B_mem_ cells reached in the *intermediate phase* persisted for many years thereafter, into the *maintenance phase* (Figure 3).

### Age related differences in specific IgG levels

To be able to determine if age impacts the dynamic range of the pertussis specific humoral compartment, Ptx, FHA, and Prn specific IgG levels in various phases after clinical infection were compared for under-fours, schoolchildren, adolescents, adults and (pre) elderly. In general, all age-groups had higher GM IgG levels for all antigens in the acute phase than in the *maintenance phase*. These differences were all significant, except for the under-fours with regard to all antigens, and the (pre-) elderly regarding FHA and Prn (Figure 4). Detected age effects concerned primarily the under-fours in the acute phase. The children in this cohort had significantly lower Ptx and FHA specific IgG levels than the older participants (Figure 4). For Prn the same trend was visible but this was not significant. After the *acute phase*, age no longer influenced IgG levels, except for the (pre) elderly who had significantly higher FHA specific IgG levels compared to the under 4′s and the adolescent group in the *maintenance phase*. Noteworthy, in the *maintenance phase*, adolescents tended to have lower IgG levels for Ptx and Prn than schoolchildren; however this was only significant for Prn.

### Age related differences in specific B_mem_ frequencies

Then we studied whether age plays a role in the size range of the pertussis specific cellular compartment by comparing frequencies of Ptx, FHA, and Prn specific B_mem_ cells in the five age groups. Again, for each age-group responses in the three *time after exposure* phases were compared. Comparable to the specific IgG response, in all age-groups highest GM levels of pertussis specific B_mem_ cells were generally exhibited in the acute phase, though significance of differences with maintenance levels was not reached for Ptx in any age group, nor for FHA and Prn for under-fours and schoolchildren (Figure 5). The B_mem_ cell response showed an age dependent effect that was also consistent with the IgG response; age affected maximum levels predominantly in the *acute phase*. More generically than the specific IgG response, detected levels of circulating specific B_mem_ cells increased not only when comparing under-fours with all other age-groups, but along the whole life-span until adulthood (Figure 5). Specific B_mem_ cell levels remained detectable for all antigens for many years after exposure and this was age-independent.

### Correlations between antigen specific memory B_mem_ cells and circulating IgG antibodies

Levels of circulating specific IgG antibodies were analysed for their correlation with frequencies of corresponding B_mem_ cells. Assessing all data from all subjects collectively, the highest correlation was found for FHA, followed by Ptx and lastly Prn, as is shown in Figure 6. When stratified according to *time after exposure* we observed that the humoral and cellular compartments seemed interconnected to some degree, albeit not equally for all antigens in all phases (data not shown). A moderate correlation between IgG levels and B_mem_ cell frequencies was found for FHA in the *acute phase* (Spearman ρ = 0.58, p<0.001) as well as in the *maintenance phase* (Spearman ρ =  0.47, p<0.01), but not in the *intermediate phase*. For Prn, a moderate correlation was found only in the acute phase (Spearman ρ =  0.47, p<0.001). Ptx on the other hand showed no correlation between humoral and cellular B cell responses in the *acute phase* (Spearman ρ =  0.19, p<0.09), but a weak correlation in the in the *maintenance phase* (Spearman ρ =  0.37, p<0.002).

### Longitudinal data of specific B_mem_ and IgG levels

**Figure 7:**
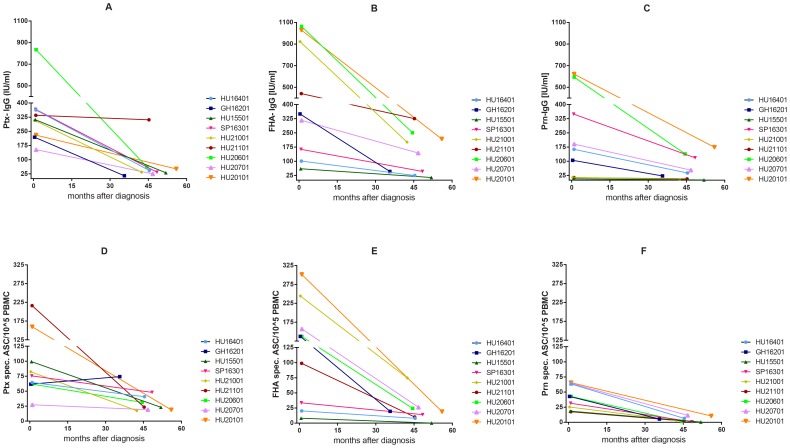
Longitudinal analysis of levels of specific IgG and B_mem_ cells in nine pertussis patients. Antigen specific IgG levels at two time points per patient for Ptx (A), FHA (B) and Prn (C) and corresponding B_mem_ frequencies for Ptx (D), FHA (E) and Prn (F) are depicted.

To verify whether the trends related to closeness to infection found per *time after exposure* phase in the cross-sectional analysis can also be observed on an individual basis, we determined levels of Bp specific IgG and B_mem_ cells in two longitudinal samples from nine individuals (Figure 7). The first sample was taken in the *acute phase,* on average 0.8 months [range: 0.4–1.0] after laboratory confirmed diagnosis of a symptomatic pertussis episode, and the second in the *maintenance phase*, on average 46 months [range: 36–56] after diagnosis (see table 2 for details). In general, the longitudinal data confirmed our cross-sectional data; both specific IgG levels and specific B_mem_ levels were higher at the first time-point than the second, for all three measured pertussis antigens. As an exception, one individual presented comparable IgG concentrations against Ptx and FHA at the second time point as in the acute phase, while the corresponding B_mem_ cell frequencies of this subject steeply declined. In contrast, another subject showed an increase in the Ptx specific B_mem_ cell response between the first and the second sampling point, while the corresponding level of IgG declined. In line with the cross-sectional data, highest specific IgG and B_mem_ cell levels in the nine individuals were found for FHA, followed by Ptx and lastly by Prn. Overall, we observed a considerable individual variation in the *acute phase*, and a more moderate individual variation in the *maintenance phase*.

## Discussion

In the present study dynamics of pertussis specific B cell responses were investigated for the first time in various age-groups comprising symptomatic (ex) pertussis patients. Both the impact of time since last exposure to antigen and age on quantitative serological and cellular parameters of B cell immunity were studied. The analysis of Bp specific responses yielded three main outcomes. Firstly, the size of the specific B_mem_ cell population is considerably inflated after a pertussis infection and declines thereafter. This is comparable to the decay of pertussis specific IgG levels, yet with antigen specific differences. The levels of Ptx specific IgG decline fast compared to those of FHA and Prn, while levels of Ptx specific B_mem_ cells decline more slowly when compared to FHA and Prn specific counterparts_._ Post-infection waning of Ptx-IgG levels has been described before [51]–[53]. We found both cellular and humoral immune parameters wane to low but detectable maintenance levels, within 9 months after antigen encounter. The longitudinal serological and B_mem_ cell data obtained from nine subjects confirmed our cross-sectional analysis. Studies researching pathogen specific B_mem_ cell levels in large cohorts after natural exposure are limited. Pinna *et al* showed a progressive decrease of varicella zoster virus specific B_mem_ cells over a period of 17 years in one individual [54]. Alam *et al* compared cholera specific B_mem_ cell responses in naturally infected patients to responses in healthy vaccinated adults and found highest levels of cholera toxin B subunit specific B_mem_ cells at day 30 for both naturally infected subjects and vaccinees. However, a decrease to baseline level occurred fastest in vaccinees [55]. Studies investigating B_mem_ cell responses to human malaria parasites determined that *Plasmodium falciparum* can induce long lasting B_mem_ that are maintained for several years without re-exposure [56], [57]. For pertussis the size of the B_mem_ cell population has mostly been investigated in vaccination studies in children by Buisman and co-workers [38], [40], [58]. Nine year olds, 5 years after the Dutch preschool (aP) booster, still showed GM levels of 3.2, 14.2 and 4.0 specific ASC when using purified B cells [40] and 1.6, 6.2 and 2.5 specific ASC per 10^5^ PBMC for Ptx, FHA and Prn respectively, when PBMC were used as in our B ELIspot assay (Annemarie Buisman personal communication). These levels are comparable to the FHA specific levels of 8.0 ASC per 10^5^ PBMC and somewhat lower than the Ptx and Prn specific ASC of 5.7 and 5.1 ASC per 10^5^ PBMC respectively in our age group of infected schoolchildren in the *maintenance phase*. Whether natural infection truly induces higher levels of pertussis specific B_mem_ compared to vaccination needs further investigation.

Secondly, weak to moderate correlations were found between pertussis specific B_mem_ cell levels and corresponding serological values in an antigen-dependent manner. The strongest correlation was found for FHA, followed by Ptx and Prn. When analysed per *time after exposure* phase we found a moderate correlation in the *acute phase* for FHA and Prn, but not for Ptx. In the *maintenance phase* a correlation was found for FHA and Ptx but not for Prn. For none of the antigens a correlation was found in the *intermediate phase*. There is as yet no consensus in literature on whether levels of specific antibodies should correlate with corresponding levels of B_mem_ cells. In various antigen models correlations were described directly after immunisation [56], in others only in steady state conditions [59], [60]. Pre-existing B_mem_ cells will respond to renewed pathogen encounter in various ways. One part of B_mem_ cells will directly differentiate into short-lived plasma blasts, responsible for a fast anamnestic IgG response. Another part will (re) enter into a germinal centre reaction, leaving either as new B_mem_ cell progeny or as new long-lived plasma cells providing the long term IgG response [35], [61]–[65]. It is unknown what drives the relative ratio of these alternative fates. However, our finding of (weakly) correlating sizes of Bp specific humoral and B_mem_ cell compartments in the *acute* or *maintenance phase* after a natural infection confirms the notion that both types of recall responses have a common denominator, most likely the size of the pre-existing B_mem_ cell population.

Thirdly and most noteworthy, we found that age impacted the dynamic range of pertussis specific B cell responses. Our flow cytometry data confirmed age-related shifts in overall B cell populations. With increasing age, CD19^+^ B cells frequencies in lymphocytes declined, and the proportion of CD19^+^CD27^+^ B_mem_ cells within the CD19^+^ B cell population increased, as described by several other studies [47]–[50]. Our study is unique regarding the large number and wide age range of symptomatic pertussis (ex) patients. We could thus uncover that age determines the maximum, but not the base-line size of B_mem_ populations. Higher levels of B_mem_ cell specific for Ptx, FHA and Prn, and to some extend this was also true for IgG, were reached when comparing older with younger age-groups in the acute phase, whereas not in the *intermediate* or *maintenance phase*. Hence at younger age in the *acute phase*, a lower frequency of specific B_mem_ cells was established, and in under-fours a lower peak level of specific IgG was seen. Our data suggest that the quantitative Bp specific B cell response is confined differently at the extremes of life. For post-infection Ptx-serology, a tendency towards a higher peak and faster decline with increasing age was earlier noted by Versteegh *et al* [51]. Various explanations can be considered to account for age effects in our data. The limited peak levels of Bp IgG and B_mem_ cell populations in the under-fours could reflect immatureness of their specific B cell compartment. These young children could also be poor responders to pertussis antigens, and therefore have become ill despite vaccination, as described by Taranger *et al* [66]. The vaccine type of the primary series the under-fours received was an acellular pertussis vaccine (aP) whereas the other vaccinated groups had largely received whole-cell pertussis vaccine (wP). Hendrikx et al found that aP priming results in higher post-vaccination levels of pertussis specific IgG and B_mem_ cells than wP priming [39], [67]. Since our results show lowest B_mem_ levels in the aP primed under-fours, other factors than primary series must play a role in the age-effect. A more relevant factor seems to be the number of encounters with antigen, serially increasing with age. In steady state phase, Hendrikx *et al* found higher levels of Bp specific IgG and numbers of B_mem_ cells in six and nine year olds, 2 and 5 years after their last vaccination, than in three and four year olds, 2 and 3 years after their last vaccination, respectively [38]. Apart from age, the difference between the former and latter groups of children was a booster vaccination at 4 years. Also, later in life, the higher peak levels of circulating specific B_mem_ cells found in adults and (pre) elderly could be caused by more encounters with circulating Bp, each exposure producing a higher level of B_mem_ cells than the previous one. However, these higher B_mem_ cell levels did not co-occur with the capacity to raise higher peak levels of IgG. A larger B_mem_ cell compartment at higher age does not necessarily indicate more effective B cell immunity. Blomberg and coworkers showed that elderly have a reduced ability to generate high affinity antibodies and IgG1 and IgG3 responses against influenza A hemagglutinin [68], [69]. Failure to produce high affinity antibodies could also account for a prolonged expansion of the B_mem_ cell compartment, due to the absence of negative feedback of immune complexes on B cell proliferation [70]. A study interrogating such qualitative aspects of B cell responses in our age cohorts is currently on-going. FHA is a component of Bp shared with other *Bordetellae* and other pathogens (e.g. *H. influenzae*, *M. pneumoniae* [71], [72]). Therefore for FHA, higher GM levels of specific B_mem_ cells found in the *maintenance phase* in (pre) elderly compared to younger age-groups could be the result of cross-reactivity. Also, its relatively large molecular size and number of potential B cell epitopes could further add to the recruitment of higher numbers of specific B_mem_ cells compared to the other pertussis antigens. This makes FHA a strong sensor to detect cohort effects in B cell responses.

While we largely attribute the established differences between the age-cohorts to biological age or encounters with live pathogens, we also observed an effect of the pre-school aP booster vaccination only received by the cohort of schoolchildren. This group had generally higher GM IgG levels than the older group of adolescents. For the three studied pertussis antigens this difference reached only significance for Prn, pointing out that Prn is perhaps the most immunogenic vaccine antigen. The downside of this could be the emergence of Prn-deficient *B. pertussis* strains that is currently on-going in countries which have a Prn containing aP vaccine [16], [17], [73]. Knowing that *B. pertussis* is still circulating and that protective immunity to pertussis after vaccination wanes relatively fast, it is clear that symptomatic and asymptomatic infections will keep occurring throughout life. To obtain a longer duration of immunity to pertussis new vaccines will have to be developed. Studies increasing our knowledge on age related quantitative and qualitative trends in specific B cell memory responses are hereby imperative.
